# High toxinogenic potential of *Staphylococcus aureus* from wild ungulates in Brandenburg, Germany with a low level of antibiotic resistance

**DOI:** 10.3389/fvets.2024.1445413

**Published:** 2024-07-23

**Authors:** Tobias Lienen, Rafael Hernán Mateus-Vargas, Julia Steinhoff-Wagner, Martin H. Richter, Sven Maurischat

**Affiliations:** ^1^Department Biological Safety, German Federal Institute for Risk Assessment (BfR), Berlin, Germany; ^2^Department Safety in the Food Chain, German Federal Institute for Risk Assessment (BfR), Berlin, Germany; ^3^Department of Animal Sciences, University of Göttingen, Göttingen, Germany; ^4^TUM School of Life Sciences, Animal Nutrition and Metabolism,Technical University of Munich, Freising-Weihenstephan, Germany

**Keywords:** *Staphylococcus aureus*, wild ungulates, virulence, antimicrobial resistance, phylogeny

## Abstract

**Introduction:**

Data regarding the occurrence and virulence of *Staphylococcus* (*S*.) *aureus* in wild living animals is rare. However, *S. aureus* may carry a multitude of virulence factors and express resistance to several antimicrobial substances. Handling game meat may thus lead to serious infections or food poisoning. The aim of this study was to provide insights into the occurrence and characteristics of *S. aureus* in wild ungulates from Brandenburg, Germany.

**Methods:**

Nasal swabs of externally healthy-looking wild boars, roe, fallow and red deer were collected in hunts during season 2021/2022 and analyzed for *S. aureus* by selective enrichment. Species were determined using matrix assisted laser desorption ionization mass spectrometry and tested for phenotypic antimicrobial resistance. Whole-genome sequencing was conducted for genotyping, determination of virulence associated genes and analysis of phylogenetic relationships.

**Results:**

*S. aureus* were recovered from approximately 8% of nasal swabs. However, the strains were only obtained from the sampled wild ruminants. *S. aureus* isolates were associated with sequence types (ST) 1, ST30, ST133, ST425, ST582 and ST6238. Isolates of ST1 clustered closely together in the phylogenetic analysis. Genes encoding staphylococcal enterotoxin (SE) or SE-like (SEl) were found in 14/17 isolates. In particular, a *seh* gene was present in 12/17 isolates. Moreover, two isolates harbored a multiplicity of genes encoding SE or SEl. In addition, the toxic shock syndrome toxin encoding *tst* gene was detected in one isolate. This isolate was resistant to penicillin and cefoxitin and accordingly harbored the *blaZ* gene.

**Discussion:**

Wild ungulates intended for human consumption may carry potentially virulent *S. aureus*. In one case, the close phylogenetic relationship of *S. aureus* isolates indicates a possible intraspecific spread within a common territory. However, for others, the origin or the spread pattern can only be inferred. Handling of animals or their carcasses might contribute to staphylococcal infections in humans. Moreover, food poisoning due to SE producing strains may occur, if recommended hygiene practices are not applied during processing of game meat.

## 1 Introduction

*Staphylococcus* (*S*.) *aureus* is a coccoid formed and Gram-positive bacterium that inhabits a wide variety of organs and tissues, such as the skin and mucosa of animals and humans. *S. aureus* may carry a large number of virulence associated genes; however, animals and humans may be colonized without showing disease symptoms. The ability to evade the human immune system as well as its capacity to express leucocidins and further toxins makes it to one of the most harmful bacteria for human and animal health. For instance, infections with *S. aureus* may lead to wound infections in all mammalian species, mastitis in dairy herds, skeletal disorders in poultry, or even a sepsis in humans (1–3). Moreover, due to the zoonotic potential of *S. aureus*, a transmission from animals to humans or vice versa may occur. In particular, the production of staphylococcal enterotoxin (SE) by *S. aureus* is a significant threat with regard to food poisoning. Thereby, the classical SE types A to E have the highest potential to cause food poisoning (4). Ingestion of SE contaminated food may lead to nausea, vomiting, diarrhea or abdominal pain. Besides SEs, the toxic shock syndrome toxin is of high importance for human health when being infected with *S. aureus* (5). Concerning antimicrobial resistance, methicillin-resistant *S. aureus* (MRSA) is of great concern for animal and human health, since treatment options may fail during an infection with MRSA. MRSA are resistant to virtual all beta-lactam antibiotics and often carry resistances to more antimicrobial classes. The beta-lactam antibiotic resistance in MRSA is most commonly transmitted by the *mecA* gene, however, in wildlife species such as wild hedgehogs, rabbits, and rodents, the *mecC* gene is also frequently found (6–9).

Data regarding *S. aureus* in wildlife is still rare in comparison to livestock or companion animals. *S. aureus* was found in a multiplicity of wild animal species such as rat, hedgehog, beaver, squirrel, fox, wild boar, deer or even bat, elephant and dolphin (10, 11). Also different MRSA clones were detected in a multitude of wildlife samples, expressing the potential of wild animals to act as reservoir and sentinel for MRSA carriage (12). Several studies have been conducted with regard to the genotypic characterization of *S. aureus* in wild animals. Thereby, a large variety of sequence types and clonal complexes was detected (10, 13). Moreover, *S. aureus* from wildlife and game meat was shown to exhibit the potential to produce virulence factors such as Panton-Valentine leucocidin (14, 15) or staphylococcal enterotoxins (16, 17). According to the European Union One Health 2022 Zoonoses Report, *S. aureus* enterotoxins were the second most frequently reported bacterial toxin-producing agents in foodborne outbreaks, and first for the number of hospitalizations and deaths in the year 2022 (18).

In Germany, game meat from wild boar, fallow, red and roe deer is frequently consumed and its handling may possess a possible risk for *S. aureus* transmission to humans. However, only few studies were conducted so far with respect to the *S. aureus* occurrence in wild animals that are hunted for human consumption. Therefore, the objective of this study was to investigate the occurrence of *S. aureus* in wild ungulates hunted in the German Federal State of Brandenburg. Furthermore, the presence of genes associated with virulence as well as antimicrobial resistance (AMR) was determined by whole-genome sequencing and bioinformatics.

## 2 Materials and methods

### 2.1 Sample collection and isolation

Sampling activities were conducted within a framework agreement involving the German Federal Institute for Risk Assessment (BfR) and the German Institute for Federal Real Estate (BImA). For details regarding the sampling strategy, please refer to Maaz et al. (19). In total, 23 driven hunts were visited in 16 different districts during the hunting season 2021/2022 from October 2021 to January 2022 in Brandenburg, Germany. Hunting was conducted several times per week. Two hundred and fifteen nasal swab samples of wild boars (*n* = 78), roe (*n* = 84), fallow (*n* = 38) and red deer (*n* = 15) were collected (Table 1). The samples were transported to the laboratory on day of collection and stored at 4°C until further analysis. Swab samples were analyzed for *S. aureus* by selective enrichment using the ISO 6888-3:2003 method (20), which is in particular applied when expecting low numbers of *S. aureus*. This protocol includes an initial enrichment step in modified Giolitti-Cantoni broth (Merck Millipore, USA) with addition of a 1% potassium tellurite solution (Merck Millipore, USA) under anaerobic conditions and subsequent streaking on Baird-Parker agar plates (Oxoid, UK) including egg yolk tellurite emulsion (Oxoid, UK). According to differences in colony morphologies, a loop of one to three presumptive *S. aureus* colonies was transferred to sheep blood agar plates (Oxoid, UK) and incubated for 20–24 h at 37°C. Species were determined using matrix assisted laser desorption ionization mass spectrometry (MALDI-TOF-MS; Bruker, USA). Colonies were spotted on the MALDI-TOF-MS target via direct transfer method and covered with 0.8 μl of α-Cyano-4-hydroxycinnamic acid (Bruker Scientific LLC). According to the manufacturer’s recommendations, the threshold score for acceptable *S. aureus* species identification was ≥2.000. The reference database was provided by Bruker (MBT-BDAL-8468).

**Table 1:** Number of sampled animals and detection rate of *S. aureus* with respect to the hunting districts in Brandenburg, Germany.

Hunting district	Sampled animals				Total animals per district	Detection of *S. aureus*	Detection rate
	Fallow deer	Red deer	Roe deer	Wild boar			
A	-	1	6	8	15	-	0%
B	-	-	6	4	10	-	0%
C	2	-	5	11	18	-	0%
D	1	-	3	1	5	1 (RoD)	20%
E	-	6	7	7	20	1 (RD)	5%
F	-	4	2	7	13	-	0%
G	9	-	-	1	10	-	0%
H	-	1	8	5	14	-	0%
I	-	-	3	-	3	-	0%
J	-	2	2	5	9	-	0%
K	-	-	2	2	4	1 (RoD)	25%
L	-	-	18	6	24	-	0%
M	-	-	6	3	9	-	0%
N	22	1	5	10	38	12 (11 FD 1 RD)	32%
O	4	-	3	1	8	1 FD	13%
P	-	-	8	7	15	1 (RoD)	7%
Total	38	15	84	78			

RoD, roe deer; RD, red deer; FD, fallow deer.

### 2.2 Whole-genome sequencing and bioinformatic analysis

All isolates that were identified by MALDI-TOF-MS as being *S. aureus* were inoculated in 5 ml brain-heart-infusion broth and incubated at 37°C for 24 h. DNA of 1 ml culture was extracted using the Qiagen DNeasy Blood and Tissue Kit (Qiagen, Germany) according to the manufacturer’s protocol modified by adding 10 μl lysostaphin (Sigma Aldrich, USA) to the lysis buffer. The DNA library was prepared using an Illumina DNA Prep kit (Illumina Inc., USA) and the 150 bp paired-end sequencing run was performed on an Illumina NextSeq 500 instrument. Raw Illumina reads were trimmed and *de novo* assembled with the in-house developed AQUAMIS pipeline (21). Bacterial characterization such as determination of sequence types and analysis of AMR and virulence associated genes was done with the in-house developed Bakcharak pipeline.^1^ Phylogenetic analyses using core genome multi-locus-sequence-typing (cgMLST) as well *spa* type determination were conducted using Ridom SeqSphere+ version 7.0.4 according to the *S. aureus* cgMLST scheme comparing 1861 alleles (22). *Spa* types were additionally verified by PCR analysis and subsequent sanger sequencing (Eurofins, Luxembourg).

### 2.3 Antimicrobial susceptibility testing

Antimicrobial susceptibility testing was performed at the National Reference Laboratory for Antimicrobial Resistance at the BfR by broth microdilution according to international guidelines [ISO 20776-1:2006 (23)/CLSI M07 Ed 12 (24)]. It was carried out using a commercial standardized antibiotic panel (Sensititre EUST scheme from Thermo Fisher Scientific, UK) that is recommended by the European Food Safety Authority (EFSA) for resistance monitoring in MRSA from livestock and food (25). For interpretation of the minimum inhibitory concentration (MIC) of the individual strains, the following EUCAST ECOFFs for *S. aureus* (accessed 2024-06-11) were used: Penicillin >0.125 mg/L; Cefoxitin >4 mg/L; Chloramphenicol >16 mg/L; Ciprofloxacin >2 mg/L; Clindamycin >0.25 mg/L; Erythromycin >1 mg/L; Fusidic acid >0.5 mg/L; Gentamicin >2 mg/L; Kanamycin >8 mg/L; Linezolid >4 mg/L; Mupirocin >1 mg/L; Rifampin >0.016 mg/L; Streptomycin >16 mg/L; Quinupristin-Dalfopristin >1 mg/L; Tetracycline >1 mg/L; Tiamulin >2 mg/L; Trimethoprim >2 mg/L; Vancomycin >2 mg/L. For quality control of resistance testing, the *S. aureus* strain ATCC 29213 and *Enterococcus faecalis* strain ATCC 29212 were used.

## 3 Results

### 3.1 Occurrence and genotypic characteristics of *S. aureus* in wild ungulates

*S. aureus* was recovered from 17/215 nasal swabs in six of the 16 hunting districts (D, E, K, N, O, P) by selective enrichment. This corresponds to a total detection rate of approximately 8% among the sampled artiodactyl species. The positive rate per district was 32% (N), 25% (K), 20% (D), 13% (O), 7% (P) and 5% (E; Table 1). Most isolates were retrieved from fallow deer (*n* = 12; 32% positive rate), whereas fewer isolates were retrieved from red (*n* = 2; 13% positive rate) and roe deer (*n* = 3; 4% positive rate; Table 1). *S. aureus* was not found in nasal swabs from wild boars in the respective hunting season. *S. aureus* isolates were associated with *spa* types t127 (*n* = 10), t1872 (*n* = 1), t693 (*n* = 2), t3043 (*n* = 1), t3583 (*n* = 1), t085 (*n* = 1), and t472 (*n* = 1). Sequence types (ST) 1 (*n* = 12), ST30 (*n* = 1), ST133 (*n* = 1), ST425 (*n* = 1), ST582 (*n* = 1), and ST6238 (*n* = 1) were determined (Table 1).

### 3.2 Phylogenetic relationships

**Figure 1:**
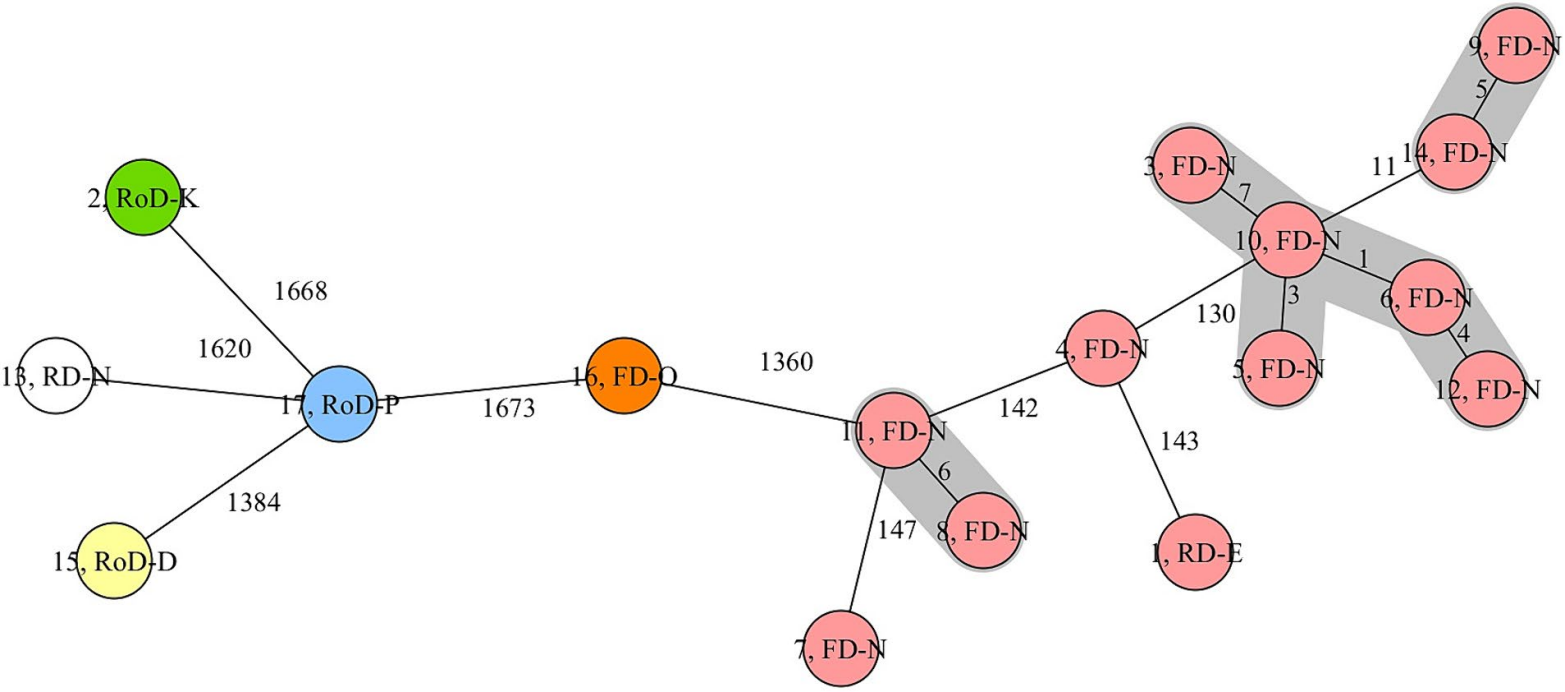
Phylogenetic analysis (core genome multilocus sequence typing) of *S. aureus* isolates with various sequence types (colours) from different wild ungulates (1–17) and districts (letters). FD, fallow deer; RD, red deer; RoD, roe deer. Red, ST1; Orange, ST582; Blue, ST425; Green, ST30; Yellow, ST133; White, ST6238.

The cgMLST analysis showed that t127-ST1 isolates of fallow deer (No. 3, 4, 5, 6, 7, 9, 10, 12, 14) obtained from hunting district N generally had less than 150 allelic differences (AD). The isolates from other districts were, despite of one case, considerably different based on genomic analysis. As shown in Figure 1, a cluster was built of five closely related t127-ST1 *S. aureus* isolates (No. 3, 5, 6, 10, 12) from district N that only differed in a maximum of seven alleles. In addition, two other closely related clusters consisting of two t127-ST1 (No. 9, 14) and two t693-ST1 isolates (No. 8, 11), respectively, appeared. The additional two other t127-ST1 isolates from fallow deer (No. 4, 7) and district N were only distantly related to the t127-ST1 cluster isolates with AD >100. Interestingly, the only isolate of red deer (No. 13) obtained in hunting district N was associated to ST6238 as well as *spa* type t3043 and considerably differed from the other t127-ST1 isolates of fallow deer. In contrast and despite the geographical distance between hunting districts E and N (linear distance *ca.* 75 km), the t127-ST1 isolate (No. 1) of red deer from hunting district E had comparably lower AD to one isolate of fallow deer from hunting district N (No. 4; Figure 1).

### 3.3 Antimicrobial resistance and virulence associated genes

The number of AMR genes was rather low (Table 2). Four isolates carried the fosfomycin resistance transmitting *fosB* gene. One of these isolates additionally harbored the *blaZ* gene and expressed phenotypic resistance to penicillin (MIC >2 mg/L) and cefoxitin (8 mg/L) in the antimicrobial susceptibility testing. The isolates were susceptible to all other antimicrobial substances according to the EUCAST ECOFFs mentioned in section 2.3. This t1872-ST30 isolate was obtained from a roe deer fawn (<1 year old) sampled in a hunting district located at the periphery of the metropolitan area of Berlin-Potsdam. A high number of virulence associated genes was detected in each isolate. Genes encoding SE or SE-like (SEl) were found in 14/17 isolates (Table 2). In particular, an *seh* gene was present in all t127-ST1 isolates disregarding the hunting district of origin. The t1872-ST30 isolate harbored genes encoding SEA, SEG, SEI, SEM, SEN, SEO and SElU and the t3043-ST6238 isolate of the hunting district C carried genes for SEI, SEM, SEN, SEO and SElU. Besides SE and SE-like genes, the toxic shock syndrome toxin (TSST) encoding *tst* gene was detected in the t1872-ST30 isolate (Table 2). Moreover, the t1872-ST30 isolate carried the immune evasion cluster genes *scn*, *chp* and *sak*.

**Table 2:** Genotypic characteristics, antimicrobial resistance (AMR), and virulence associated genes in *S. aureus* isolates from wild ungulates from different districts and animals in Brandenburg, Germany.

No.	Source	District	*spa* type	ST^1^	AMR^2^ genes	AMR phenotype^3^	Virulence associated genes^4^
1	Red deer	E	t127	1	-	Susceptible	*seh*
2	Roe deer	K	t1872	30	*blaZ, fosB*	PEN, FOX	*sea, seg, sei, sem, sen, seo, seu, tst, scn, chp, sak*
3	Fallow deer	N	t127	1	-	Susceptible	*seh*
4	Fallow deer	N	t127	1	-	Susceptible	*seh*
5	Fallow deer	N	t127	1	-	Susceptible	*seh*
6	Fallow deer	N	t127	1	-	Susceptible	*seh*
7	Fallow deer	N	t127	1	-	Susceptible	*seh*
8	Fallow deer	N	t693	1	-	Susceptible	*seh*
9	Fallow deer	N	t127	1	-	Susceptible	*seh*
10	Fallow deer	N	t127	1	*-*	Susceptible	*seh*
11	Fallow deer	N	t693	1	-	Susceptible	*seh*
12	Fallow deer	N	t127	1	-	Susceptible	*seh*
13	Red deer	N	t3043	6238	-	Susceptible	*sei, sem, sen, seo, seu*
14	Fallow deer	N	t127	1	-	Susceptible	*seh*
15	Roe deer	D	t3583	133	*fosB*	Susceptible	-
16	Fallow deer	O	t085	582	*fosB*	Susceptible	-
17	Roe deer	P	t472	425	*fosB*	Susceptible	-

Only positive hunting districts are shown.

1 ST, Sequence Type.

2 AMR, Antimicrobial Resistance.

3 PEN, Penicillin (MIC > 2 mg/L); FOX, Cefoxitin (8 mg/L).

4 Selection of relevant virulence associated genes with regard to food safety and human health.

## 4 Discussion

Wild ungulates may be carriers of potentially pathogenic microorganisms such as *S. aureus*. Since game meat is frequently consumed, food safety aspects are elementary in this area. In this study, the occurrence and genotypic characteristics of *S. aureus* were determined in hunting districts of the federal forest division of the BImA in the German Federal State of Brandenburg in the hunting season 2021/22.

### 4.1 Occurrence and sequence types

In total, approximately 8% of all sampled wild ungulates carried *S. aureus* in their nostrils; however, the positive rates in fallow deer (32%) and red deer (13%) were higher than in roe deer (4%). On the contrary to other studies, which showed a prevalence of up to 18% in Spain (26), 33% in Portugal (27) or 37% in Germany (28), in this study, *S. aureus* was not found in the nostrils of wild boars in the respective hunting season.

A recent Portuguese study elucidates the epidemiological links of *S. aureus* at the animal-human interface (29). Several sequence types were determined in the detected *S. aureus* isolates in this study. Although some *S. aureus* can be classified into livestock-associated, healthcare-associated or community-associated according to their ST, other STs have been reported from various sources e. g. humans, livestock, companion animals and wildlife (30). The latter seems to be the case in this study for those being associated to ST1, ST30, ST133, ST425, ST582, and ST6238. *S. aureus* with ST1, ST30, ST133, and ST425 were already reported in different species of wild animals before (10) and their presence was also shown in humans (31). *S. aureus* ST582 seems to be more prevalent in human clinical samples (32–34) than in animal samples; however, in a previous study, ST582 was detected in a purulent subcutaneous lesion of a farm rabbit (35). *S. aureus* ST6238 is a relatively new sequence type and was originally detected in a horse in the year 2022 (36).

### 4.2 Transmission of *S. aureus* in wild ungulates

Interestingly, several t127-ST1 *S. aureus* isolates obtained from fallow deer of the same district and during the same hunt clustered closely together in the phylogenetic analysis comparing their core genomes. The close genetic relationship of the respective isolates indicates a common source of colonization, possibly accompanied by intraspecific spread, within the local fallow deer population. There may be several reasons for the clonality of the isolates. One possible hypothesis is that these wild artiodactyls might have been colonized in the same area in this specific district, where *S. aureus* inhabits a particular niche and was transmitted to the carriers. *S. aureus* may for instance be present in surface water (37) or being secreted via animal feces (38, 39), so that a transmission may take place in close contact to the contaminated area; e.g. during sniffing or rolling on the ground. However, neither of the ten wild boars nor the five roe deer sampled within the same hunting district carried *S. aureus*, which would contradict this assumption. A further possibility is that positive individuals with clonal *S. aureus* isolates might be related to each other. Fallow deer are gregarious animals, often forming herds of does accompanied by fawns and few males during various seasons (40). ST1 isolates were obtained primarily from fawns and young fallow deer (<2 years) and therefore could have been transmitted from one animal to another due to close contact within the herd. The need of close contact may be further supported by the notable difference to the *S. aureus* isolate of the red deer sampled at the same hunting district. It is also noteworthy that European herds of fallow deer usually stay within a relatively small territory (in average 3 to 5 km^2^) compared to European red deer [in average 9 to 36 km^2^; (41)]. Some red deer populations additionally have long migration routes (42, 43). Thus, the infection of this red deer individual may have occurred under other habitat conditions. Moreover, the distant relation of *S. aureus* isolates from different districts illustrates that, at least in the sampled areas, there was no obvious transmission of *S. aureus* between the districts, probably due to the long distances between hunting districts or to local habitat fragmentation (44).

### 4.3 Antimicrobial resistance and virulence factors

*S. aureus* is one of the high priority pathogens with regard to annual deaths and antimicrobial resistance (45). Especially, the most prevalent ST1 in this study has also been frequently reported as MRSA in either a clinical, human context, in which the strains often harbor the staphylococcal chromosome cassette (SCC) *mec* type IV as well as virulence genes for human adaptation like the immune evasion cluster genes *sak* and *scn,* or in a livestock context harboring the SCC*mec* V in combination with resistance genes *erm*(A) and *vga*(A) (46, 47). In contrast, the ST1 isolates in this study lack all of these typical genes for human or livestock adaptation and seem to be unaffected from antimicrobial pressure. Only the t1872-ST30 *S. aureus* isolate in this study showed phenotypic resistance to the beta-lactam antibiotics penicillin and cefoxitin, whereas the other isolates were susceptible to all tested antimicrobials. Accordingly, the t1872-ST30 isolate harbored the beta-lactamase coding *blaZ* gene, which confers resistance to penicillin and, in the case of hyperproduction, may also confer resistance to oxacillin or cefoxitin (48, 49). Interestingly, this strain was obtained from an individual hunted in a district near Berlin and Potsdam, which is among the areas with the higher settlement coverage of all hunting districts regularly sampled by the BfR (41). Moreover, the isolate was obtained from a roe deer fawn. As reviewed before (41), this individual likely did not roam long distances before sampling, as European females of this deer species typically have a territory size <1 km^2^. Thus, it is highly probable that transmission occurred directly from the immediate environment or the mother doe. Except the t1872-ST30 isolate, all other *S. aureus* isolates of this study were susceptible to the tested antimicrobial substances and did not harbor any AMR genes despite the fosfomycin resistance transmitting *fosB* gene discovered in four isolates. A recent study shows an increasing prevalence of the *fosB* gene in *S. aureus* (50). The high susceptibility to most antimicrobials seems to be reasonable, since antimicrobial pressure in the hunting areas predominantly covered by forest may be low, there is no need for bacteria to develop or keep antimicrobial resistance to survive. However, wild ungulates living in regions more intensively used for livestock farming were observed to carry *Staphylococcaceae* isolates with reduced susceptibility to cefoxitin more often than wild ungulates in less anthropized areas (41, 51). Despite these observations and coincidences, we deliberately refrain from delving further into the possible transmission routes and sources of the sampled animals due to the general lack of epidemiological information in this field. Future studies should focus on providing more detailed insights into possible transmission routes in anthropized habitats.

*S. aureus* may carry a multiplicity of virulence associated genes. With respect to food safety, in particular staphylococcal enterotoxin or staphylococcal enterotoxin-like genes are of great importance (4). The uptake of SEs via food such as milk or meat may lead to nausea, vomiting, diarrhea or abdominal pain. Especially young and old people or persons with immunodeficiencies are prone to suffer severely from staphylococcal food poisoning. In this study, 14/17 isolates harbored SE or SEl genes. In particular, an *seh* gene was detected in all of the t127-ST1 *S. aureus* isolates and is highly conserved in strains of this ST. (47) Although SEH is regarded as less potent than other SEs, food poisoning by SEH due to consumption of contaminated food was occasionally reported (52, 53). Two isolates carried a multiplicity of SE or SEl genes in their genomes in this study. The t3043-ST6238 *S. aureus* isolate was characterized by genes for SEI, SEM, SEN, SEO and SElU. Those genes are located in the so-called enterotoxin gene cluster (egc) (54). Food poisoning caused by *S. aureus* that carried the *sei* gene was already described in previous studies (55, 56). However, SEI seems to have a rather low emetic activity (57) and *sei* carrying strains only produce a relative low amount of SEI (58). However, isolates carrying the *egc* were shown to be the causative agent of food poisoning and were already related to food-borne outbreaks (59). The highest toxicogenic potential was detected in the t1872-ST30 *S. aureus* isolate in this study. ST30 *S. aureus* are characterized as human-adapted strains (60) and was isolated from a comparably strong anthropized area. Besides its antimicrobial resistance, as mentioned above, this isolate carried genes encoding a high number of staphylococcal enterotoxins as well as genes for the toxic shock syndrome toxin and the immune evasion cluster. Elements of the immune evasion cluster function as evading mechanism with respect to the human immune system and the toxic shock syndrome toxin may act as superantigen in the human body (5). Regarding food safety, the t1872-ST30 *S. aureus* isolate in this study also carried the egc [SEG, SEI, SEM, SEN, SEO, SElU; (54)]. Moreover, the t1872-ST30 isolate harbored the *sea* gene. The appearance of *sea* and *tst* genes in t1872-ST30 *S. aureus* is common (61). The *sea* gene is often located within the immune evasion cluster complex (62). SEA is the most common toxin in relation to food poisoning (63) and several food-borne outbreaks caused by SEA were reported (64, 65).

The finding of potentially virulent *S. aureus* in wild ungulates elucidates that during handling of hunted animals as well as processing of game meat strict hygienic measures are of significant importance to prevent direct transmission of *S. aureus* from animals to humans or meat surfaces, and thus the possibility of serious infections and food poisoning. This study is limited to wild ungulates from the German Federal State of Brandenburg. However, this data expands the knowledge about zoonotic pathogens in specific regions in Germany.

## 5 Conclusion

Wild animals may carry potentially virulent *S. aureus* as indicated by the findings of this study. The close phylogenetic relationship of *S. aureus* isolates indicates a possible spread between animals from the same territory or a common source of colonization. Handling of animals or their carcasses might contribute to staphylococcal infections in humans. Moreover, meat contamination and subsequent food poisoning due to SE producing strains may occur, if recommended hygiene measures are not applied during processing game meat. Monitoring of pathogens in wild game animals is of great importance to evaluate pathogen ecology, zoonotic potential, significant risks and develop recommendations.

## Data availability statement

The datasets presented in this study can be found in online repositories. The names of the repository/repositories and accession number(s) can be found at: https://www.ncbi.nlm.nih.gov/bioproject, PRJNA641762.

## Ethics statement

Ethical approval was not required for the study involving animals in accordance with the local legislation and institutional requirements because the basis of the sampling approach was a framework agreement involving the German Federal Institute for Risk Assessment (BfR) and the German Institute for Federal Real Estate (BImA). The federal forest division of the BImA is responsible for forest and game management over 476,000 ha of hunting area in Germany.

## Author contributions

TL: Conceptualization, Data curation, Funding acquisition, Investigation, Methodology, Writing – original draft, Writing – review & editing. RM: Conceptualization, Data curation, Writing – original draft, Writing – review & editing. JS-W: Conceptualization, Writing – original draft, Writing – review & editing. MR: Conceptualization, Writing – original draft, Writing – review & editing. SM: Conceptualization, Funding acquisition, Writing – original draft, Writing – review & editing.

## Footnotes

^1^https://gitlab.com/bfr_bioinformatics/bakcharak
